# Structural neuroanatomy of human facial behaviors

**DOI:** 10.1093/scan/nsae064

**Published:** 2024-09-23

**Authors:** Fate Noohi, Eena L Kosik, Christina Veziris, David C Perry, Howard J Rosen, Joel H Kramer, Bruce L Miller, Sarah R Holley, William W Seeley, Virginia E Sturm

**Affiliations:** Department of Neurology, University of California, San Francisco, CA 94158, United States; Department of Neurology, University of California, San Francisco, CA 94158, United States; Department of Neurology, University of California, San Francisco, CA 94158, United States; Department of Neurology, University of California, San Francisco, CA 94158, United States; Department of Neurology, University of California, San Francisco, CA 94158, United States; Department of Neurology, University of California, San Francisco, CA 94158, United States; Department of Psychiatry and Behavioral Sciences, University of California, San Francisco, CA 94158, United States; Department of Neurology, University of California, San Francisco, CA 94158, United States; Department of Psychiatry and Behavioral Sciences, University of California, San Francisco, CA 94158, United States; Department of Psychiatry and Behavioral Sciences, University of California, San Francisco, CA 94158, United States; Department of Psychology, San Francisco State University, San Francisco, CA 94132, United States; Department of Neurology, University of California, San Francisco, CA 94158, United States; Department of Neurology, University of California, San Francisco, CA 94158, United States; Department of Psychiatry and Behavioral Sciences, University of California, San Francisco, CA 94158, United States

**Keywords:** facial behavior, emotion, cingulate cortex, primary motor cortex, supplementary motor area

## Abstract

The human face plays a central role in emotions and social communication. The emotional and somatic motor networks generate facial behaviors, but whether facial behaviors have representations in the structural anatomy of the human brain is unknown. We coded 16 facial behaviors in 55 healthy older adults who viewed five videos that elicited emotions and examined whether individual differences in facial behavior were related to regional variation in gray matter volume. Voxel-based morphometry analyses revealed that greater emotional facial behavior during the disgust trial (i.e. greater brow furrowing and eye tightening as well as nose wrinkling and upper lip raising) and the amusement trial (i.e. greater smiling and eye tightening) was associated with larger gray matter volume in midcingulate cortex, supplementary motor area, and precentral gyrus, areas spanning both the emotional and somatic motor networks. When measured across trials, however, these facial behaviors (and others) only related to gray matter volume in the precentral gyrus, a somatic motor network hub. These findings suggest that the emotional and somatic motor networks store structural representations of facial behavior and that the midcingulate cortex is critical for generating the predictable movements in the face that arise during emotions.

## Introduction

Humans have expressive faces. Despite the importance of the face in emotions and social communication (Ekman 1993, Ekman and Rosenberg 2005, Keltner et al. 2019a), many questions remain about how the brain controls the facial musculature (Rinn 1984, Westbrook et al. 2021). The emotional and somatic motor systems are distributed networks that can produce targeted changes in the dozens of muscles that comprise the human face (Holstege et al. 1996). The emotional motor system has hubs in the midcingulate cortex (MCC) and anterior cingulate cortex (ACC) and tight connections with the amygdala, hypothalamus, and periaqueductal gray (Devinsky et al. 1995, Parkinson et al. 2009, Caruana et al. 2015, 2018). The somatic motor system, in contrast, is anchored by the precentral gyrus and premotor cortex and projects to the spinal and bulbar motor nuclei via the pyramidal tract (Holstege and Subramanian 2016). The supplementary motor area (SMA) and facial motor nucleus (Kuypers 1958, Morecraft et al. 2004, 2017, Vogt 2009, Pan et al. 2012, Gothard 2014) participate in both systems (Jürgens 1984, Holstege et al. 1996, Nguyen et al. 2014). See Supplementary Fig. S1.

Much of what is known about the roles of the emotional and somatic motor networks in facial motor control comes from research in other species. Tracer studies in nonhuman primates have revealed mappings of the face in brain regions that correspond to human anterior MCC (aMCC), posterior MCC, precentral gyrus, premotor cortex, SMA, and facial motor nucleus (Morecraft et al. 2001, 2004). The structural organization of these regions sheds light on how these networks produce precise and predictable facial movements. In each region, there is some evidence for a somatotopic organization such that neurons that produce similar movements in the body are clustered together (Mitz and Wise 1987, Godschalk et al. 1995, Morecraft et al. 1996, Raos et al. 2003, Cauda et al. 2011, Procyk et al. 2016, Loh et al. 2018, Roux et al. 2020). The extent to which the neurons that project to different facial muscles have a discrete or intermingled organization in these regions, however, remains a topic of ongoing discussion. In the facial motor nucleus, where the lower motor neurons that innervate the facial muscles reside, there is some evidence of somatotopy as neurons that innervate similar sets of facial muscles are arranged together in longitudinal columns (Komiyama et al. 1984, Jenny and Saper 1987, Choi and Raisman 2002, Furutani et al. 2004, Morecraft et al. 2004).

In humans, fine-grained neuroanatomical maps of the facial musculature are lacking. Electrical stimulation, functional neuroimaging, and lesion studies have revealed clear representations of the head and face in the human precentral gyrus (Penfield and Boldrey 1937, Roux et al. 2020, Gordon et al. 2023), SMA (Allison et al. 1996), and ACC/MCC (Caruana et al. 2018), but little is known about the structural and functional representations of specific facial movements. One electrical stimulation study, which examined the facial movements that result from stimulation of specific brain regions, probed the precentral gyrus and revealed dissociable mappings of various parts of the face such as the forehead, eyebrows, eyes, nose, cheeks, lips, and chin (Roux et al. 2020). At the level of the facial muscles, one functional neuroimaging study uncovered distinct yet overlapping representations of four voluntary facial movements in the precentral gyrus (Krippl et al. 2015). Investigations of posed facial behaviors, however, cannot shed light on the anatomical basis of involuntary facial movements such as those that arise during emotions (Levenson 1999, Matsumoto et al. 2008, Cowen et al. 2021).

While both the emotional and somatic motor networks may contribute to human facial behavior, how each system supports the generation of emotional facial behaviors is not well understood. Initial clinical studies found people with damage in the face area of the precentral gyrus could not move their facial muscles on command but could spontaneously exhibit facial behavior during emotions (Monrad-Krohn 1924, Hopf et al. 1992, Töpper et al. 1995, 2003, Trepel et al. 1996). Complementary studies have shown that people with damage in the MCC, ACC, and SMA exhibit altered emotional facial behavior but spared voluntary facial behavior (Hopf et al. 1992, Sturm et al. 2008, Eckart et al. 2012). Taken together, these studies suggested that the emotional and somatic motor networks operate independently. In subsequent studies, a more nuanced picture has emerged that includes reciprocal connections between the emotional and somatic networks at the cortical (Morecraft and van Hoesen 1992, Jezzini et al. 2015, Gerbella et al. 2016) and subcortical (Jenny and Saper 1987, Holstege 2002, Holstege et al. 2003) levels. Interactions between these networks are important for numerous functions. For example, connections between the facial motor nucleus and the periaqueductal gray modulate the facial muscles during vocalization (Holstege and Subramanian 2016), and connections between SMA with aMCC contribute to motor planning (Nguyen et al. 2014). In sum, while the emotional and somatic motor networks can each operate without input from the other, activity in one system can influence activity in the other (Vaca et al. 2011, Caruana et al. 2015, 2018).

In previous human research, it has been challenging to delineate the roles of the emotional and somatic motor networks in facial motor control because few studies include detailed neuroimaging and facial behavior measures. Electrical stimulation studies can examine the neural mechanisms underlying facial behavior, but these studies are rare, invasive, and usually rely on qualitative descriptions of facial muscle movements (Penfield and Boldrey 1937, Caruana et al. 2018, Roux et al. 2020). Functional neuroimaging studies investigating the neural correlates of facial behavior have only examined volitional facial movements (Krippl et al. 2015) or a single spontaneous emotional facial behavior (i.e. smiling; Iwase et al. 2002). Challenges with quantifying facial behavior also abound. Manual facial coding systems, which rely on human raters, remain the gold standard for measuring facial behavior (Dupré et al. 2020). Coding facial behavior in any context—even outside the scanner—is laborious because facial movements are dynamic (i.e. muscles contract and relax over time) and interdependent (i.e. activation of certain muscles can alter the appearance of other muscles). Thus, manual coding systems require significant training, and it takes a substantial amount of time to code even limited quantities of behavioral data (Ekman et al. 1994, 2002). Taken together, these methodological challenges have limited research efforts to localize the representations of specific facial movements in the brain.

In the present study, we investigated the structural neuroanatomy of human facial behaviors. We used an objective coding system to quantify facial behavior in healthy older adults who watched a series of emotionally evocative film clips. Given that brain structure and function are tightly connected (Pang et al. 2023), we conducted structural neuroimaging analyses to identify the brain regions in which gray matter volume correlated with specific facial behaviors. As older adults have developed and refined their face–brain connections through a lifetime of emotional expression and social communication, a healthy aging sample may be ideal for mapping associations between facial behavior and gray matter volume. First, we examined whether participants who displayed greater emotional facial behavior during specific trials had larger gray matter volume in the MCC, a hub within the emotional motor system that plays a critical role in emotion generation (Iwase et al. 2002, Vogt 2016). Next, we investigated whether participants who displayed more facial behavior in general (across trials) had larger gray matter volume in the face area of the precentral gyrus, a key region in the somatic motor network (Morecraft et al. 2004). Just as experienced musicians who practice complex finger movements have greater gray matter volume in the hand region of the precentral gyrus than those who play less often (Gärtner et al. 2013), we hypothesized that participants who were more expressive overall would have larger gray matter in this region.

## Materials and methods

### Participants

Fifty-five healthy older adults (mean age = 74.0 years, SD = 4.3 years, 62% female; Table 1) were recruited from the University of California, San Francisco (UCSF) Hillblom Healthy Aging Network. Participants were volunteers recruited from the community who underwent an extensive multidisciplinary evaluation that included a clinical history, neurological examination, neuropsychological testing, informant-based interview of daily functioning, and structural magnetic resonance imaging (MRI). The participants had no past or current neurological or psychiatric disorders, and they did not have mild cognitive impairment or dementia. Participants provided informed written consent before completing the study, which was approved by the UCSF Human Research Protection Program.

**Table 1.** Demographic information and neuropsychological measures.

	Mean (SD)
*N*	55
Age (years)	74 (4.3)
Sex: % female	62%
Education (years)	17.4 (1.8)
Handedness: % right-handed	94%
Mini-mental state examination (/30)	29.3 (0.9)
California Verbal Learning Test-II (16-word list)	11.7 (3.4)
Benson figure copy 10-minute recall (/17)	11.3 (2.1)
Modified trails (correct lines per minute)	39.5 (15.6)
Modified trails errors	0.2 (0.6)
Phonemic fluency (# correct in 60 s)	16.1 (4)
Semantic Fluency (# correct in 60 s)	21.9 (4.4)
Design fluency correct (# correct in 60 s)	12 (3.5)
Digits backward	5.4 (1.3)
Boston naming test spontaneous correct (/15)	14.7 (0.5)
Benson figure copy (/17)	15.5 (0.7)

Demographic information and neuropsychological test results are shown for the sample. Mean and standard deviation (SD) are provided. Two participants did not complete all the neuropsychological tests.

### Laboratory assessment of emotion

#### Procedure

Participants were seated in a comfortable chair, which was placed 4.25 feet away from a computer monitor (21.5 inches). A remote-controlled, semi-obscured video camera recorded the testing session; participants were notified of the camera during the informed consent procedure prior to testing. Participants received instructions about the overall structure of the testing session and completed a battery of tasks designed to assess various aspects of emotion; only the emotional reactivity task was analyzed in the present study.

#### Emotional reactivity task

Participants watched a series of five emotion-inducing film clips. Prior to each film clip, participants sat quietly for a 60-s pretrial baseline period during which they looked at a black “X” on a white screen. Each film clip was chosen to elicit a specific emotion (awe, sadness, amusement, disgust, or nurturant love) and lasted approximately one and a half minutes. The awe film clip showed images of nature that showcased the vastness of universe (from “Planet Earth”); the sadness film clip showed a hospital scene in which a mother learned her family was in a car accident (from “21 Grams”); the amusement film clip showed a baby laughing (“Baby Ripping Paper” video from YouTube); the disgust film clip showed the removal of ear wax from the ear canal (“Ear Wax” video from YouTube); and the nurturant love film clip showed human babies interacting with baby animals (from “Babies Around The World”). All participants watched the film clips in the same order. Piloting in a separate sample of healthy adults and results from our previous studies have confirmed these videos elicit the target emotions (Sturm et al. 2021).

#### Measures

##### Subjective experience

After each film clip, there was a 30-s post-trial period during which participants saw an “X” on the screen. To assess subjective experience, participants then rated the degree to which they felt a variety of emotions (i.e. awe/amazement, nurturant love/affection, amusement/happiness, excitement/enthusiasm, embarrassment, pride, surprise, anger, sadness, disgust, and fear) while watching each film clip. They reported whether they experienced each emotion “not at all,” “a little,” or “a lot.”

##### Facial behavior

We used the Dynamic Affective Facial Action Coding System (DFACS), a coding system based on the Facial Action Coding System (FACS), which we developed to simplify and expedite our manual facial coding process (see Supplementary material). In DFACS and FACS, movements in the face are referred to as action units (AUs), and each AU reflects the observable contraction of one or more underlying facial muscles (e.g. AU 12 refers to a facial movement in which lip corners are pulled upward into a behavior typically recognized as a smile). The activation of an AU can occur alone or in combination with other AUs and may or may not signal the presence of an emotion (Ekman et al. 1994, 2002). A team of four FACS-certified coders (including C.V., who was part of the coding team led by S.R.H.) who were blind to the study design, hypotheses, and stimuli content used Noldus Observer XT software (version 14) to rate the activity of 16 emotion-relevant AUs (Supplementary Table S1) in participants during the most intense 30 s of each film clip (as determined by an independent group of raters prior to coding).

The videos of the participants (which were recorded at a speed of 30 frames per second) were coded in a continuous manner over three passes at a slow speed (e.g. one-fifth time). Coders hit designated keyboard keys to indicate when activity in each AU started, stopped, or changed intensity. They rated the activity in each AU on a four-point scale that included intensities of 0 (“absent”), 1 (“slight but noticeable”), 2 (“moderate”), and 3 (“strong”). AU 25 (lips parted) was only coded as 0 (“absent”) or 1 (“present”). The coding data were later exported from the Noldus Observer software system in 1-s intervals. If the coder indicated an AU was active for most of the 1-s interval, the intensity score for the AU during that 1-s interval was exported; if the AU was active for less than half of the 1-s interval, a 0 was exported.

Each participant’s videos were randomly assigned to one member of the coding team who served as the “primary coder,” and most of the videos (75%) were also assigned to a second coder who served as its designated “reliability coder.” The second-by-second scores for each AU were entered into a confusion matrix, and inter-observer agreement was quantified with coefficient kappa, which is the proportion of agreement above what would be expected to occur by chance (Cohen 1960, Fleiss 1981). This method is recommended for calculating the reliability of observer-based measures of facial behavior (Cohn et al. 2007); coefficients of 0.60–0.75 indicate good or adequate reliability, and coefficients of ≥0.75 indicate excellent reliability (Fleiss 1981). The coders’ inter-observer reliability for all codes across all videos was excellent (Cohen’s kappa = 0.81) (Cohen 1960), a level surpassing that of existing automated classifiers (Dupré et al. 2020). To ensure reliability estimates remained high when including intensity scores, we reanalyzed the coders’ reliability using the Reliability Analysis feature within the Noldus Observer software. The software generated a confusion matrix assessing the time-based intersection of two coders’ data, with the additional benefits of (I) improving the temporal resolution of the codes (i.e. codes were analyzed every hundredth of a second) and (II) evaluating the degree of agreement between observers’ intensity codes. Kappa values were obtained for all double-coded videos; the average kappa value across all videos remained excellent (Cohen’s kappa = 0.78).

### Neuroimaging acquisition and preprocessing

#### Acquisition

Participants underwent 3 Tesla MRI using a TIM Trio scanner (Siemens, Iselin, NJ, USA) at the Neuroscience Imaging Center of the UCSF. The structural T1 images were acquired using a 12-channel head coil (160 sagittal slices, slice thickness: 1.0 mm, field of view: 256 × 230 mm^2^, matrix: 256 × 230, voxel size: 1.0 × 1.0 × 1.0 mm^3^, repetition time: 2300 ms, time to echo: 2.98 ms, flip angle: 9°). The MRI scans were completed within 18 months of participants’ laboratory assessment of emotion.

#### Preprocessing

Prior to any preprocessing steps, we visually inspected the T1 images to exclude poor quality scans. No participants were excluded based on visual inspection. We used the Computational Anatomy Toolbox version 12 (CAT12) in MATLAB (version R2018b) to conduct a homogeneity check using the Mahalanobis distance (Pierna et al. 2002), which uses the weighted overall image quality and mean correlation of images to identify scans with the highest deviations from the sample’s average. This comprehensive quality check suggested that no scans were outliers in the sample, and thus, no participants warranted exclusion. Next, the images were segmented into gray matter, white matter, and cerebrospinal fluid. The gray matter maps were normalized to Montreal Neurological Institute (MNI) space, modulated, and smoothed with an 8-mm Gaussian kernel. These preprocessed gray matter images were then used in the linear regression analyses.

### Data reduction and statistical analyses

#### Subjective experience and facial behavior

To confirm that the film clips elicited the target emotions, we examined participants’ subjective experience and facial behavior. For each trial, we quantified the percentage of participants who endorsed feeling each emotion at the different intensity levels. We next conducted Spearman’s correlation analyses (Spearman 1904) to assess whether participants who reported more intense subjective experience also displayed more intense facial behavior. The data were analyzed using RStudio statistical software version 3.5.3 (RStudio: Integrated Development for R, PBC, Boston, MA, USA, URL: http://www.rstudio.com/).

#### Neuroimaging analyses

To examine the neural correlates of facial behavior, we conducted voxel-based morphometry (VBM) analyses (Ashburner and Friston 2000) in MATLAB (version R2018b). First, we examined emotional facial behavior displayed “within” certain trials. Here, we focused on the disgust and amusement trials because they typically elicit strong and distinct facial movements (Eckart et al. 2012, Chen et al. 2020, Sturm et al. 2021). As there can be some variability in the facial behaviors that unfold during emotions (Cowen et al. 2021), we examined the facial behaviors with the highest total activity scores in addition to prototypical behaviors, as defined by prior studies (Cordaro et al. 2018, Cowen et al. 2021). To compute these measures of within-trial emotional facial behaviors, we averaged the second-by-second intensity scores for each AU in each trial (over 30 s) and then summed these scores for AUs of interest. We next focused on facial behavior displayed “across” the trials. We calculated a total activity score for each AU by averaging its second-by-second intensity scores across all five trials (over 150 s). We then summed these scores across AUs to obtain a single measure of total facial behavior (Supplementary Table S2).

In each VBM analysis, we ran a linear regression to examine whether facial behavior predicted voxel-wise gray matter volume in regions of interest while controlling for age, sex, and total intracranial volume (i.e. the sum of gray matter, white matter, and cerebrospinal fluid, which is an index of head size). Nonhuman primate studies have mapped the musculotopic organization of the facial muscles in several brain regions by injecting anterograde tracers (into brain) and retrograde tracers (into facial muscles) and following their projections (Morecraft et al. 2001, 2004). These studies have found multiple areas that represent the face (Vogt and Pandya 1987, Vogt 2016) in the precentral gyrus (primary motor cortex), ventrolateral premotor cortex, SMA, aMCC (extending into pregenual ACC), and posterior MCC. We used the Brainnetome atlas (https://atlas.brainnetome.org) and MARSBAR toolbox (https://marsbar-toolbox.github.io/) to create a combined mask of these regions for each hemisphere (Supplementary Fig. S2).

We used Statistical NonParametric Mapping (version 13) software (Nichols and Holmes 2002; http://warwick.ac.uk/snpm). Results were considered significant at *P *< .005, uncorrected, because in neuroimaging studies with the sample sizes of <200, stricter statistical thresholds lead to more inflated and less reliable results (Marek et al. 2022). Nonparametric approaches, which are common in neuroimaging studies, make minimal assumptions about the probability distribution (Holmes et al. 1996, Nichols and Holmes 2002) and, thus, are more suitable for small samples. Control over type I error due to multiple comparisons was accomplished by conducting 10 000 permutations, which is the recommended number for VBM analyses (Dickie et al. 2015). Using this method in combination with *a priori* masks minimized the likelihood of spurious findings. Results are also reported at *P *< .05 when surviving family-wise error correction (FWE). For each regression analysis, the resulting maps were overlaid on the MNI template using MRIcroGL software (v1.0.20180623).

### Replication study

To confirm the validity of our results, we conducted additional neuroimaging analyses in an independent sample of healthy adults (*N *= 60) who completed a structural brain MRI and had the same facial coding data available from a similar emotional reactivity task. This sample allowed us to test whether our results were robust across different cohorts and tasks. See Supplementary material for details.

Although there are no prior structural neuroimaging studies of human facial behaviors, as a final test, we also compared our results with a prior human electrical stimulation study (Roux et al. 2020). Roux et al. (2020) found stimulation of certain areas in the precentral gyrus elicited movements in the face (forehead, eyebrows, eyes, nose, cheek, and jaw). Within the precentral gyrus, the mean coordinates for these facial movements were *x* = −53.5, *y* = −2.7, and *z* = 36.9. We used the MARSBAR toolbox to create a 10-mm sphere around the peak coordinates reported in Roux et al. (2020) and plotted our results alongside their findings.

## Results

### Videos elicited subjective experience and facial behavior

Throughout the task, participants reported a range of subjective experience (Supplementary Fig. S3) and displayed a variety of facial behaviors. Across the trials, 87% of the 16 coded AUs were activated at least once, with intensities ranging from mild to strong (Supplementary Table S3).

During the disgust trial, 73% of participants reported experiencing moderate to high disgust (33% reported “a lot” and 40% reported “a little”). AUs 4 (brow furrowing) and 6/7 (eye tightening) had the highest total activity scores during the disgust trial (Supplementary Table S3); thus, we computed a total activity score for AUs 4 and 6/7 for this trial. In addition, we considered other prototypical emotional behaviors. As nose wrinkling and upper lip raising (AUs 9 and 10) are common facial movements that often accompany disgust (Matsumoto et al. 2008, Lucey et al. 2010), we also computed a total activity score for these AUs during the disgust trial. Greater disgust experience during this trial was not associated with greater activity in AU 4, *rho* = 0.002, *P *= .984, but greater experience of disgust, surprise, and awe/amazement (the most intensely endorsed emotions in this trial) correlated with greater activity across AUs 4, 6/7, 9, and 10, *rho *= 0.29, *P *= .032.

During the amusement trial, 100% of participants reported experiencing moderate to high amusement (88% reported “a lot” and 12% reported “a little”). AUs 12 (lip corners pulling up) and 6/7 (eye tightening) had the highest total activity scores during the amusement trial (Supplementary Table S3); thus, we computed a total activity score for AUs 12 and 6/7 for this trial. Given that smiling and eye tightening are the prototypical facial behaviors that arise during amusement (Cordaro et al. 2018, Cowen et al. 2021), we did not calculate any additional measures. The positive association between amusement experience and AU 12 activity during this trial approached statistical significance, *rho *= 0.26, *P *= .051. Greater subjective experience of amusement/happiness, love/affection, and excitement/enthusiasm (the most intensely endorsed emotions in this trial) correlated with greater activity across AUs 12 and 6/7, *rho *= 0.30, *P *= .023.

### MCC and SMA volumes correlate with emotional facial behaviors within trials

**Figure 1.**
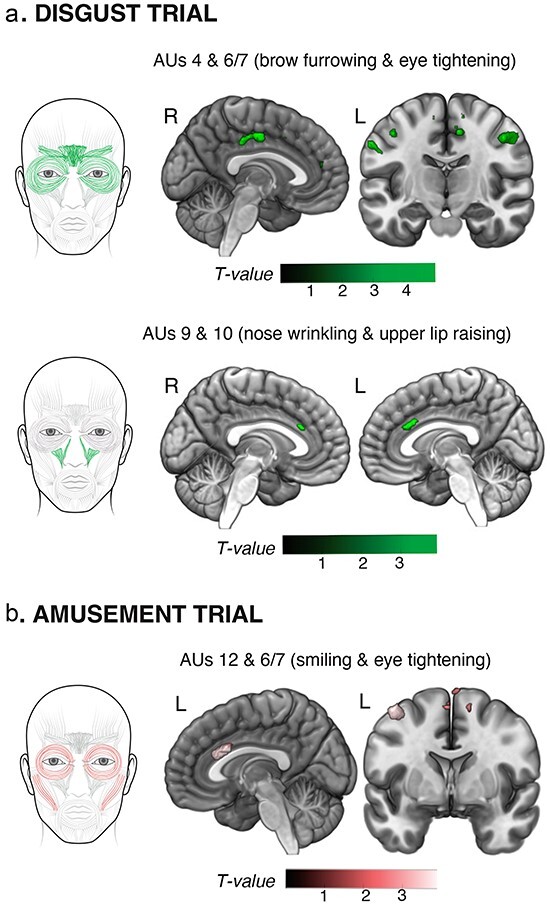
MCC represents emotional facial behaviors during specific trials. (a) Brow furrowing (AUs 4 and 6/7) during the disgust trial had a predominant representation in the right posterior MCC and bilateral precentral gyrus. Nose wrinkling and upper lip raising (AUs 9 and 10) were associated with bilateral aMCC. (b) Like nose wrinkling and upper lip raising during the disgust trial, smiling (AUs 12 and 6/7) during the amusement trial had a predominant representation in the left aMCC and bilateral SMA. The color bars display the *T*-scores at *P *< .005, uncorrected. Adobe Illustrator software was used to create the faces by manually tracing the anatomical drawing of facial muscles in the FACS manual, MRIcroGL software was used to illustrate the results on brain slices, and Affinity Designer was used to refine the final figure.

At the most stringent statistical threshold (*p*_FWE_ < 0.05), neuroimaging analyses revealed that greater total brow furrowing and eye tightening (AUs 4 and 6/7) during the disgust trial correlated with larger gray matter volume in the right posterior MCC. At uncorrected levels (*P *< .005), these facial movements also correlated with gray matter volume in bilateral precentral gyrus (Fig. 1a, Table 2). Greater nose wrinkling and upper lip raising (AUs 9 and 10) during the disgust trial, in contrast, were associated with greater gray matter volume in bilateral aMCC (Fig. 1a, Table 2). Greater total smiling and eye tightening (AUs 12 and 6/7) during the amusement trial correlated with larger gray matter volume in the left aMCC (in a region that overlapped with the cluster associated with nose wrinkling and upper lip raising in disgust) as well as in the right SMA (Fig. 1b, Table 2).

**Table 2.** Neural correlates of brow furrowing and nose wrinkling in the disgust trial and smiling in the amusement trial.

				MNI coordinates		
	Side	Cluster size	*T*-score	*x*	*y*	*z*
Disgust trial						
AUs 4 and 6/7						
Posterior MCC*	R	91	3.89	12	−10	46
Precentral gyrus	R	118	3.67	48	−8	42
Precentral gyrus	L	58	3.14	−62	2	33
Posterior MCC	R	31	2.94	10	−28	39
Precentral gyrus	L	17	2.93	−52	−9	51
AUs 9 and 10						
aMCC	L	79	2.98	−2	26	26
aMCC	R	14	2.80	0	26	26
Amusement trial						
AUs 12 and 6/7						
SMA	R	27	3.37	14	−8	63
aMCC	L	211	3.11	−8	18	27

Structural neuroimaging analyses revealed the neuroanatomical regions that had a positive association with AUs 4 and 6/7 and AUs 9 and 10 in the disgust trial and AUs 12 and 6/7 in the amusement trial (controlling for age, sex, and total intracranial volume). MNI coordinates given for the maximum *T*-score for each cluster (*P *< .005, uncorrected, *k* > 10).

* Denotes significance at *p*_FWE_ < 0.05.

### Precentral gyrus volumes correlate with facial behaviors across all trials

**Figure 2.**
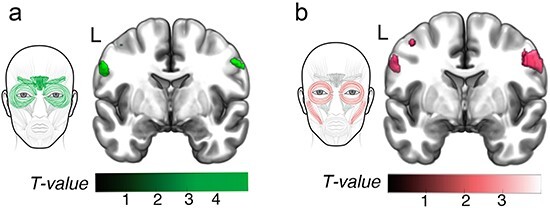
Neural correlates of total brow furrowing and total smiling across trials. Across trials, (a) total brow furrowing (AUs 4 and 6/ 7) and (b) total smiling (AUs 12 and 6/7) had predominant neural representations in bilateral primary motor cortex. These analyses confirmed that the AU combination total activity, when measured across trials rather than a specific emotional context, was represented in the primary motor cortex, a pattern that was also found for total facial behavior (across trials). The color bars display the *T*-scores at *P *< .005, uncorrected. Adobe Illustrator software was used to create the faces by manually tracing the anatomical drawing of facial muscles in the FACS manual, RStudio software was used to generate the boxplots, MRIcroGL software was used to illustrate the results on brain slices, and Affinity Designer was used to refine the final figure.

Our findings indicated that MCC and SMA were critical for producing emotional facial behaviors that arose in specific trials but also suggested that the precentral gyrus was critical for representing movements in the face. We next investigated whether a different pattern would emerge when we examined the neural correlates of these same facial behaviors but removed their connection to an emotion-inducing trial. Unlike nose wrinkling and upper lip raising (AUs 9 and 10), which were only activated during disgust trial, brow furrowing (AUs 4 and 6/7) and smiling (AUs 12 and 6/7) were activated during all five trials. Thus, we computed total activity scores for brow furrowing (AUs 4 and 6/7) and smiling (AUs 12 and 6/7) across all five trials and found greater facial behavior had correlates in the precentral gyrus but not in MCC (*P* < .005). Participants who displayed greater total brow furrowing and eye tightening (AUs 4 and 6/7) and greater total smiling and eye tightening (AUs 12 and 6/7) across trials had larger gray matter volume in the face area of the bilateral precentral gyrus (Fig. 2, Table 3).

**Table 3.** Neural correlates of total brow furrowing and total smiling across trials.

				MNI coordinates		
All trials	Side	Cluster size	*T*-score	*x*	*y*	*z*
AUs 4 and 6/7						
Precentral gyrus	L	116	3.52	−62	2	33
Precentral gyrus	R	54	3.06	56	−2	38
AUs 12 and 6/7						
Precentral gyrus	R	222	3.36	57	−3	38
Precentral gyrus	L	108	3.21	−57	−2	32
Precentral gyrus	L	22	2.89	−44	−14	52

Structural neuroimaging analyses revealed regions in which larger gray matter volume was associated with greater total brow furrowing (AUs 4 and 6/7) and greater total smiling (AUs 12 and 6/7) across trials (controlling for age, sex, and total intracranial volume). MNI coordinates provided for each cluster’s maximum *T*-score (*P *< .005, uncorrected, *k* > 10).

**Figure 3.**
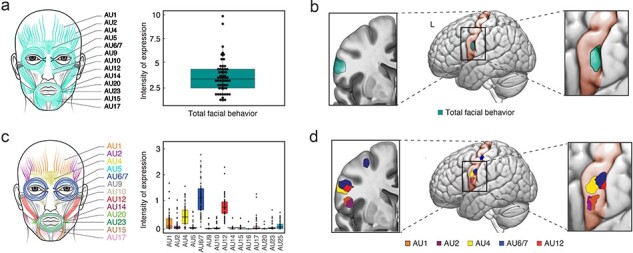
Facial behavior across trials had predominant structural correlates in the precentral gyrus. (a) For each participant, we computed a total facial behavior score by summing the single AU activity scores of all AUs across trials. (b) Greater total facial behavior across trials (all AUs combined) was associated with larger gray matter volume in the left precentral gyrus. (c) For each participant, we computed a single AU activity score for each AU by summing its average activity across all trials. (d) Greater single AU activity score across trials was associated with larger gray matter volume in distinct yet overlapping regions in the bilateral precentral gyrus. For illustration purposes, the clusters (*P *< .005, uncorrected) without the *T*-score color gradient are overlaid on the precentral gyrus in the left hemisphere. Adobe Illustrator software was used to create the faces by manually tracing the anatomical drawing of facial muscles in the FACS manual, RStudio was used to generate the boxplots, MRIcroGL was used to overlay the results on the brain slices, and Affinity Designer was used to refine the final figure.

A similar result emerged when we created a measure of total facial behavior, which represented the total activity in all AUs that a participant displayed across all five trials. Like the analyses of brow furrowing and smiling across trials, greater total facial behavior was also associated with larger gray matter volume in bilateral precentral gyrus (Fig. 3a and b, Table 4). There were no associations between total facial behavior and gray matter volume in MCC or SMA at *P* < .005. When we deconstructed the total facial behavior score to examine whether total activity in single AUs across trials also revealed a similar pattern, we found that greater total activity in AUs 1, 2, 4, 6/7, and 12 was also associated with larger gray matter volume in bilateral precentral gyrus (Fig. 3c and d, Table 4). Of these analyses, the results for AU 12 withstood correction for multiple comparisons (*p*_FWE_ < 0.05).

**Table 4.** Neural correlates of facial behavior across trials.

				MNI coordinate		
All trials	Side	Cluster size	*T*-score	*x*	*y*	*z*
Total facial behavior (all AUs combined)						
Precentral Gyrus	L	128	3.32	−57	−2	32
Precentral Gyrus	R	37	2.99	58	−2	39
AU 1						
Precentral gyrus	L	92	3.49	−58	12	21
Precentral gyrus	R	72	3.24	56	−2	22
Precentral gyrus	L	11	2.98	−54	8	3
AU 2						
Precentral gyrus	L	225	3.41	−56	8	16
Precentral gyrus	R	14	2.90	54	−3	21
AU 4						
Precentral gyrus	L	57	2.91	−60	3	30
AU 6/7						
Precentral gyrus	L	100	3.13	−62	0	34
Precentral gyrus	R	108	3.12	54	−3	40
Precentral gyrus	L	50	3.04	−44	−14	52
AU 12						
Precentral gyrus*	R	208	4.09	63	−3	30
Precentral gyrus	L	18	2.85	−56	−3	30

Structural neuroimaging analyses revealed that greater total facial behavior and greater single AU activity across trials (controlling for age, sex, and total intracranial volume) correlated with larger gray matter volume in the primary motor cortex. MNI coordinates are provided for the maximum *T*-score for the cluster (*P *< .005, uncorrected, *k* > 10).

* Denotes significance at *p*_FWE_ < 0.05.

### Replication studies

**Figure 4.**
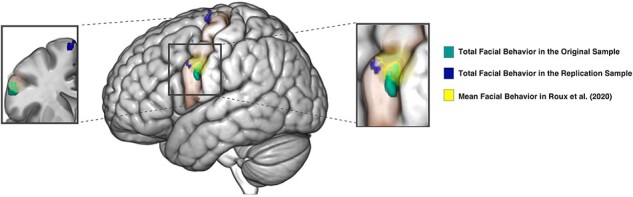
Three studies provide convergent evidence that facial behavior has structural correlates in the face area of precentral gyrus. Our original and replication samples found total facial behavior correlated with gray matter volume in the precentral gyrus. These clusters both overlapped with an area of the left precentral gyrus in which electrical stimulation elicits facial behavior in humans (Roux et al. 2020). For illustration purposes, the clusters (*P *< .005, uncorrected) without the *T*-score color gradient are overlaid on the left precentral gyrus. MRIcroGL was used to overlay the results on the brain, and Affinity Designer was used to refine the final figure.

To test the reproducibility and robustness of our original results, we took two additional steps. First, we conducted a VBM analysis in an independent replication sample of 60 healthy adults (*P *< .005, *k* > 10). Consistent with our original findings, this analysis indicated that participants with greater total facial behavior across the trials of an emotional reactivity task had larger gray matter volume in the right precentral gyrus (*T *= 3.44, 40, 4, 28), the left SMA (*T *= 3.35, −8, −10, 70), and the left precentral gyrus (*T *= 3.32, −56, 6, 38). At this statistical threshold, the bilateral clusters in the precentral gyrus were near the area of the precentral gyrus that correlated with total facial behavior across trials in our original analyses (Fig. 4).

Second, we overlaid the clusters that related to total facial behavior in our original and replication studies with the mean coordinates of facial behavior as reported in the electrical stimulation study by Roux et al. (2020). These comparisons also found convergent evidence that total facial behavior had structural correlates in the face region of precentral gyrus (Fig. 4).

## Discussion

The present study offers new insights into the structural neuroanatomy of human facial behavior. During emotions, certain movements in the face are more common than others, and we first examined emotional facial behaviors in the disgust and the amusement trials that were prominent (in the present study) or prototypical (as defined by prior studies). Consistent with previous research, brow furrowing and eye tightening (AUs 4 and 6/7) had the highest total activity scores during the disgust trial, and smiling and eye tightening (AUs 12 and 6/7) had the highest total activity scores during the amusement trial. When measured during these trials, total activity in each of these AU combinations had a predominant structural correlate in MCC. Whereas greater brow furrowing and eye tightening during the disgust trial correlated with larger gray matter volume in the right posterior MCC, greater smiling during the amusement trial correlated with larger gray matter volume in the left aMCC. Larger volume in this region also related to greater nose wrinkling and upper lip raising (AUs 9 and 10) during the disgust trial, a prototypical set of facial behaviors that often characterizes disgust. When we quantified total activity in AUs 4 and 6/7 and AUs 12 and 6/7 across trials, however, a different pattern emerged. When these same facial behaviors were measured across trials—just like the measure of total facial behavior across trials—they were no longer associated with gray matter volume in MCC but instead correlated with volume in the face areas of the left and right precentral gyrus.

Our results align with prior studies that suggest MCC, through its connections with the emotional motor network, plays a central role in producing the facial behaviors that accompany emotions (Vogt 2016). As there is some variation in the facial movements that people display during emotions across contexts, we examined the behaviors that participants exhibited most often and most intensely during the disgust and amusement trials in the present study as well as prototypical behaviors that have been established by prior research (Barrett et al. 2019, Keltner et al. 2019b). When quantified within the confines of a specific emotion-inducing trial, each set of facial behaviors related to gray matter volume in MCC. The aMCC is a key hub in the emotional motor network and, consistent with its broad role in emotion generation (Vogt 2005, Caruana et al. 2018), we found aMCC gray matter volume related to two very different facial behaviors that arose during two very different emotion-inducing trials (disgust and amusement). While stimulation of aMCC can trigger a variety of affective and goal-directed behaviors, stimulation of posterior MCC can also elicit changes in experience and especially negative sensations such as feelings of vertigo and falling into a void (Caruana et al. 2015, 2018). Individuals with greater cortical thickness (Erpelding et al. 2012) and stronger neural activation (Kunz et al. 2011) in posterior MCC are also more sensitive to negative affective cues (Pereira et al. 2010).

When we no longer limited our quantification of facial behaviors to specific emotion-inducing trials, MCC volume no longer correlated with facial behavior. Instead, the precentral gyrus, a hub in the somatic motor network, emerged as a key area. Participants with greater total facial behavior had larger gray matter representations in the face regions of the precentral gyrus than those who were less expressive. Although our results were found at an uncorrected statistical threshold, we confirmed the validity of our findings by replicating our results in an independent sample and by comparing our results to those from an electrical stimulation study (Roux et al. 2020). When we examined the neural correlates of single AUs, we uncovered a distinct cluster that related to each facial movement. These clusters appeared to have a topographic organization in which functionally related movements were in closer proximity than movements that do not often occur together. For example, the neural representation of AU 1 (which raises the inner corners of the eyebrows) in the precentral gyrus was closer to AU 2 (which raises the outer corners of the eyebrows) than to AU 12 (which raises the outer corners of the lips). The precentral gyrus anchors the somatic motor network and is important for contracting the muscles in the face, an ability that is typically assessed with tasks that elicit voluntary facial movements (Iwase et al. 2002). While it is impossible to quantify the extent to which our participants displayed involuntary and voluntary facial behaviors during the emotional reactivity task, it is possible that the precentral gyrus played a role in their within-trial emotional facial behaviors as well because this area represents movement in general or the components of their response that were deliberate.

Brain structure and function are closely connected (Pang et al. 2023), and our results suggest facial behaviors that tend to co-occur have adjacent or overlapping representations in the precentral gyrus. This type of economical arrangement has been found in human functional neuroimaging (Krippl et al. 2015) and nonhuman animal studies of the structural organization of the facial motor nucleus and SMA (Mitz and Wise 1987, Vanderwerf et al. 1998, Morecraft et al. 2001), but less is known about the precentral gyrus. Previous studies have come to different conclusions regarding the degree to which specific muscles in the face and body inhabit unique or overlapping territories in the precentral gyrus (Foerster 1931, Penfield and Boldrey 1937, Cheney and Fetz 1985, Jenny and Saper 1987, Donoghue et al. 1992, Schieber and Hibbard 1993, Sanes et al. 1995, Allison et al. 1996, Lotze et al. 2000, Indovina and Sanes 2001, Alkadhi et al. 2002, Meier et al. 2008, Rathelot and Strick 2009, Krippl et al. 2015, Wang et al. 2019, Roux et al. 2020, Gordon et al. 2023). Although our results revealed dissociable peaks for each AU in the precentral gyrus, they cannot rule out the presence of a more distributed representation of facial behavior at the neuronal level. As AUs often reflect movement of more than one underlying facial muscle (Ekman et al. 1994, 2002), our study did not have the resolution to illuminate the neural correlates of individual muscles or to determine whether there are discrete or intermingled AU representations of the facial muscles at a microscopic level. It is likely that overlapping yet distinct representations of single AUs would promote nuanced, coordinated facial motor control, which is essential for human emotions and social communication. As our study was cross-sectional, we also could not determine the causal mechanisms underlying our results. Long-term (Maguire et al. 2000, Gaser and Schlaug 2003, Boyke et al. 2008, Scholz et al. 2009, Gärtner et al. 2013) and even time-limited (Driemeyer et al. 2008, Taubert et al. 2016) repetition of motor acts can increase gray matter volume and resculpt the cortical architecture of the somatic motor network, but larger baseline gray matter volume in these areas also predicts better motor outcomes (Tomassini et al. 2011, Sampaio-Baptista et al. 2014, Zuk and Gaab 2018). Future studies will be needed to investigate whether the positive correlations that we detected between facial behavior and gray matter volume reflected the sustained effects of facial behavior on brain structure or the influence of brain structure on subsequent facial behavior.

Predictable facial behaviors make emotions recognizable and suggest the presence of biological systems that produce patterned muscle movements across individuals (Ekman and Rosenberg 2005, Matsumoto et al. 2008, Ekman and Cordaro 2011, Keltner and Cordaro 2017, Cordaro et al. 2018). How the nervous system generates these facial muscle configurations is not well understood, but comparative studies have found that facial motor control systems are more elaborated in highly social species such as great apes and humans (Sherwood et al. 2003, 2004, Sherwood 2005, Dobson 2012). Our study suggests the facial behaviors that accompany emotions may be represented as “bundled” units in the MCC. Nonhuman animal studies have found abundant evidence for bundled representations of autonomic nervous system and motor activities that promote survival-relevant functions including locomotion, respiration, and cardiac cycles (Grillner 1985, Bandler et al. 2000, Saper 2002). Bundled neural representations of facial behaviors would be an efficient means by which MCC could trigger rapid, patterned movements in the face via organized projections to the facial motor nucleus and facial musculature (Holstege et al. 1977). This type of structural organization, however, would not preclude other influences from adding variability or nuance to facial behavior across different contexts (Nieuwenhuys 1996, Cattaneo and Pavesi 2014). As MCC projects to structures in the somatic motor network (Jenny and Saper 1987, Barbas and Pandya 1989, Morecraft and van Hoesen 1992, Morecraft et al. 1996, 2001, Simonyan and Jürgens 2003, Stepniewska et al. 2006), the emotional and somatic motor networks together may help to shape how facial behavior unfolds. While the precentral gyrus is critical for representing movements in general (irrespective of the timing of the movements in a sequence), the SMA provides timing information that is critical for preparing and assembling motor patterns in a specific order (Geyer et al. 2000, Tanji 2001, Doyon et al. 2009). Together, these brain systems could create a wide range of facial behaviors by producing both predictable elements and flexibility across contexts.

The present study has limitations to consider. First, focusing on healthy older adults may have increased our ability to detect associations between gray matter volume and facial behavior because our participants had decades to establish and refine brain–face associations. It is possible, however, that age-related variables influenced our results, which would limit the generalizability of our findings to younger populations. As the participants in our study underwent extensive neurological, neuropsychological, and neuroimaging assessments and were determined to be cognitively normal and free of current neurological or psychiatric disorders, it is unlikely that they were in the early stages of an age-related disorder. Future studies are needed, however, to determine whether there are any differences between older and younger adults in the associations that we found. Second, VBM analyses cannot shed light on the functional interactions among MCC subregions, SMA, and the precentral gyrus and their roles in facial behavior production. Reciprocal connections between emotional and somatic motor networks (Holstege 2002, Holstege et al. 2003) are likely critical for the generation of emotional facial behaviors, but many questions remain as to how these systems interact. Given that the structural scaffolding of the brain determines its functional architecture (Pang et al. 2023), additional research that incorporates objective measures of facial behavior with task-based or task-free functional MRI will help to advance our understanding of the network dynamics underlying facial behavior. Third, we did not examine voluntary facial behavior in our study. To capture facial behavior outside of a specific affective context, we used an aggregate measure of total facial behavior that was computed by summing all facial behavior across trials. Although our measure of total facial behavior correlated with gray matter volume in a part of primary motor cortex that has been associated with voluntary facial behavior in previous studies (Iwase et al. 2002, Krippl et al. 2015), comparing the neural correlates of voluntary versus involuntary emotion-relevant facial movements would be an important question for future research.

## Conclusion

Our findings indicate that, like other motor acts (Cerasa et al. 2017, Dempsey-Jones et al. 2019), human facial behaviors have representations in the brain’s structural anatomy. The facial behaviors that arise during emotions are time-tested motor sequences that serve adaptive functions (Ekman and Rosenberg 2005, Keltner and Cordaro 2017, Cordaro et al. 2018). Consistent with previous research, our results suggest that the MCC, together with connected structures in the emotional motor network, plays a central role in producing the facial behaviors that accompany emotions (Jenny and Saper 1987, Barbas and Pandya 1989, Morecraft and van Hoesen 1992, Morecraft et al. 1996, 2001, Simonyan and Jürgens 2003, Vogt 2016). The precentral gyrus, together with structures in the somatic network, is critical for supporting facial behaviors in general. Our results expand current neuroanatomical models of human facial behavior and suggest the MCC stores bundled representations of facial behaviors that often co-occur, an efficient way to produce patterned emotional facial behaviors that are critical for the survival of individuals and communities.

## Supplementary Material

nsae064_Supp
