# Supplementary material for: Structural neuroanatomy of human facial behaviors
Source: Soc Cogn Affect Neurosci. 2024 Sep 23;19(1):nsae064. doi: 10.1093/scan/nsae064 (PMC11492553; doi:10.1093/scan/nsae064)
Supplement: nsae064_Supp [file nsae064_supp.zip › scan-23-296-File010.docx]

Supplementary Materials for

Structural Neuroanatomy of Human Facial Behaviors

Fate Noohi, Eena L. Kosik, Christina Veziris, David C. Perry, Howard J. Rosen, Joel H. Kramer, Bruce L. Miller, Sarah R. Holley, William W. Seeley, Virginia E. Sturm*

*Correspondence to: [virginia.sturm@ucsf.edu](mailto:virginia.sturm@ucsf.edu)

Replication Study

**Materials and Methods**

*Participants*

A total of 60 additional healthy participants (mean age=58, *SD*=15.4, 60% female) were recruited from the University of California, San Francisco (UCSF**)**. This sample did not overlap with the sample that was used in our original analyses and was used to replicate our results. This replication sample included 28 older adults (mean age=70.1, *SD*=3.9, 57% female) who were participants in the Hillblom Healthy Aging Network**,** and 32 healthy adults (mean age=46.0, *SD*=12.4, 62% female) who were participants in the ARTFL-LEFFTDS Longitudinal Frontotemporal Lobar Degeneration study. While the participants from the Hillblom Healthy Aging Network were healthy older volunteers recruited from the community, those from the ARTFL-LEFFTDS Longitudinal Frontotemporal Lobar Degeneration study were healthy family member control participants. As in the original sample, participants underwent an extensive multidisciplinary evaluation that included a clinical history, neurological examination, neuropsychological testing, informant-based interview of daily functioning, and structural magnetic resonance imaging (MRI). Participants were free of past or current neurological and psychiatric disorders and did not have mild cognitive impairment or dementia. Participants provided informed written consent before completing the study, which was approved by the UCSF Human Research Protection Program.

*Laboratory Assessment of Emotion*

*Procedure.* Participants were seated in a comfortable chair, which was placed 4.25 feet away from a computer monitor (21.5 inches). A remote-controlled, semi-obscured video camera recorded the testing session; participants were notified of the camera during the informed consent procedure prior to testing. Participants received instructions about the overall structure of the testing session and completed a battery of tasks designed to assess various aspects of emotion; only the emotional reactivity task was analyzed in the present study.

*Emotional Reactivity Task.* In each trial of the emotional reactivity task, participants viewed a block of photographs that elicited a specific emotion (nurturant love, fear, awe, sadness, disgust, or amusement) or no emotion (neutral). Each block included six photographs that were displayed in the same order for 15 seconds each. Prior to start of each trial, participants sat quietly for a 90-second pre-trial baseline period during which they were asked to look at a black “X” on a white screen.

*Facial Coding.* We used the Dynamic Facial Action Coding System (DFACS) to code the first 60 seconds of the sadness, disgust, and amusement trials. Thus, each participant had a total of 180 seconds of facial coding data (as opposed to 150 in the original sample). Unlike in our original sample, AU 6 and AU 7 were coded separately in these participants, and AU25 was coded on the same intensity range of 0-3, as opposed to 0-1.

*Neuroimaging Acquisition and Preprocessing*

Participants underwent MRI scans at the Neuroscience Imaging Center of the University of California, San Francisco. The MRI scans were completed within 18 months of participants’ laboratory assessment of emotion. The structural T1 images for half of the participants were acquired using a 3.0 Tesla (Siemens, Iselin, NJ) TIM Trio with a 12-channel head coil (160 sagittal slices, slice thickness: 1.0 mm, Field of View (FOV): 256 × 230 mm^2^, matrix: 256 × 230, voxel size: 1.0 × 1.0 × 1.0 mm^3^, Repetition Time (TR): 2300 ms, Time to Echo (TE): 2.98 ms, flip angle: 9°). For the other half the T1 images were acquired using a 3.0 Tesla Prisma scanner with a 64-channel head coil (160 sagittal slices, slice thickness: 1.0 mm, Field of View (FOV): 256 × 230 mm^2^, matrix: 256 × 230, voxel size: 1.0 × 1.0 × 1.0 mm^3^, Repetition Time (TR): 2300 ms, Time to Echo (TE): 2.98 ms, flip angle: 9°). The preprocessing steps were conducted the same way as in the original sample.

*Neuroimaging Analyses*

We conducted voxel-based morphometry (VBM) analyses (Ashburner and Friston, 2000) in Matlab (version R2021b). Given the differences between the two studies in type of emotional stimuli, we considered the across-trial measures as most comparable between the two samples. We calculated a total activity score for each AU by averaging its second-by-second intensity scores across all three trials (over 180 seconds). We then summed these scores across AUs to obtain a single measure of total facial behavior. In the VBM analysis, we ran a linear regression to examine whether facial behavior predicted voxel-wise gray matter volume in regions of interest while controlling for age, sex, total intracranial volume, and scanner type. Our primary variable in these replication analyses was the total facial behavior score.

**Dynamic Affective**

**Facial Action Coding System**

**(DFACS)**

**Manual and Coding Procedures**

***Created by***

***Sarah Holley, PhD***

***Christina Veziris, BA***

***Virginia Sturm, PhD***

**Table of Contents**

[Introduction 5](#_Toc164081090)

[Training Sequence 6](#_Toc164081091)

[FACS Codes and DFACS Coding Guidelines 7](#_Toc164081092)

[Observer Set Up 12](#_Toc164081093)

[DFACS Reliability Notes – General Guidelines 14](#_Toc164081094)

[Source Material for DFACS 15](#_Toc164081095)

# Introduction

Welcome to Project EMO. This is work being conducted in collaboration with researchers in the SFSU Relationships, Emotion, and Health (REH) laboratory, directed by Sarah Holley, and the UCSF Clinical Affective Neuroscience (CAN) laboratory, directed by Virginia Sturm.

Participants visit the UCSF CAN laboratory and engage in a number of tasks. Behavioral data was collected during some of these tasks. Our job is to code the facial behaviors and body movements the participants displayed during specific parts of a task using designated behavioral coding systems.

**DFACS Coding**

The coding system described in this manual is called the Dynamic Affective Facial Action Coding System (DFACS). This system is based on the criteria established in the Facial Action Coding System (FACS). FACS is a system designed by Paul Ekman and Wally Friesen that precisely measures movements in specific facial muscles. While FACS is considered the gold standard for capturing facial movements, it is highly time intensive to use. A subsequent system, EMFACS, was designed to be a more efficient coding system that focuses on the subset of the codes most closely associated with emotional expressions. This system, however, limits coding to expressions that contain a “core” AU or a “core combination” of codes, and thus does not enable to flexibility to capture combinations of movements that occur outside of these designated expressions.

DFACS was designed based on the criteria and coding methods describe in the FACS manual (Ekman & Friesen, 1976, 1978, 2002), the EMFACS manual (Ekman, Irwin, & Rosenberg, 1994), as well as subsequent literature examining emotional expressions and evaluations of FACS codes (see Appendix 1 for notes on source materials). Changes as compared to the FACS and/or EMFACS systems include a) a more limited set of action unit codes; b) a 3-point intensity scale; c) combining codes that rely on the same muscles/can’t be reliably distinguished (e.g., 6 and 7*; 23 and 24); d) the use new technology (i.e., the Observer software) to conduct the behavioral coding in a dynamic, continuous way (e.g., rather than from static images or designated apex/event moments); and e) coding all actions seen on the face (from the limited set included in the system), regardless of whether or not they occur as part of a “core” combination (i.e., there are no co-occurrence rules).

*In subsequent versions of this manual, these two codes were separated. This was due to the conceptual utility of coding them separately. The coding team further established that we are able to reliably distinguish the signals.

**Observer XT**

Observer XT is a system or collection, management and analysis of observational data. As a coder you will be “blind” to the stimulus the participants is watching or the activity they are doing. You will be asked to not listen to any sound coming from the video until you are satisfied that all your behavioral codes are complete. Once you have fully coded all emotional expressions and body movements, and then listen with sound to comment on any vocalizations the participant makes.

# Training Sequence

**Step 1: Learn FACS**

- Must be fully certified in FACS before you can proceed

**Step 2: DFACS Training Session(s)**

- Introduction to coding system

**Step 3: Individually Code Videos using Observer**

**Step 4: Meeting for Review & Consensus of Video Coding**

- Review codes, discuss any areas of discrepancy
- Recode as needed

**Step 5: Ongoing Training**

- Repeat process above: code training videos, meet weekly to discuss

**Step 6: Individually Code Final Test Video for Reliability****

**Must reach reliability of kappa of .70 or over in order to start coding videos; if under then code and review additional training videos until reliable**

# FACS Codes and DFACS Coding Guidelines

*The table below summarizes the FACS action units (AUs) utilized in the DFACS system. All specific criteria and sample images for each AU can be found in the FACS manual (Ekman, Friesen, & Hagar, 2002).*

**Upper Face Codes**

| **AU** | **Description** | **Facial Muscle** |
| --- | --- | --- |
| No code | Neutral | *None, face should be totally relaxed.* |
| 1 | Inner Brow Raiser | *Frontalis, pars medialis* |
| 2 | Outer Brow Raiser | *Frontalis, pars lateralis* |
| 4 | Brow Lowerer | *Corrugator supercilii, Depressor supercilii* |
| 5 | Upper Lid Raiser | *Levator palpebrae superioris* |
| 6 | Cheek Raiser  **(code as 67)** | *Orbicularis oculi, pars orbitalis OR pars palpebralis* |
| 7 | Lid Tightener  **(code as 67)** | *Orbicularis oculi, pars palpebralis* |

**Lower Face Codes**

| **AU** | **Description** | **Facial Muscle** |
| --- | --- | --- |
| 9 | Nose Wrinkler | *Levator labii superioris alaquae nasi* |
| 10 | Upper Lip Raiser | *Levator labii superioris* |
| 11 | Nasolabial Deepener | *Zygomaticus minor* |
| 12 | Lip Corner Puller | *Zygomaticus major* |
| 14 | Dimpler | *Buccinator* |
| 15 | Lip Corner Depressor | *Depressor anguli oris (a.k.a. Triangularis)* |
| 16 | Lower Lip Depressor | *Depressor labii inferioris* |
| 17 | Chin Raiser | *Mentalis* |
| 20 | Lip stretcher | *Risorius w/ platysma* |
| 23 | Lip Tightener | *Orbicularis oris* |
| 24 | Lip Pressor  **(AU24, code as 23)** | *Orbicularis oris* |
| 25 | Lips part  **(AU25, code as 25, intensity 1)** | *Depressor labii inferioris or relaxation of Mentalis, or Orbicularis oris* |
| 26 | Jaw Drop  **(AU26, code as 25, intensity 2)** | *Masseter, relaxed Temporalis and internal Pterygoid* |
| 27 | Mouth Stretch  **(AU27, code as 25, intensity 3)** | *Pterygoids, Digastric* |

**Modifiers**

***Intensity:*** Each AU is assigned an intensity value when it is coded. We will use the following scale:

- 1: Slight but noticeable (in FACS, this would be a B; *trace-level A movements are not coded*)
- 2: Moderate (in FACS, this would be a C)
- 3: Strong (in FACS, this would be a D or E)

Notes:

--If an AU is present and changes in intensity, you must deactivate then reactivate the code at the new intensity.

--No intensity codes are assigned to AU00 or AU99 (cannot code).

--See special instructions above for intensity coding for AU25.

--The intensity criteria for each AU are fully defined in the FACS manual and summarized in this manual’s appendix. The criteria listed for intensity level B are the MINIMUM necessary requirements to be scored in DFACS (since trace levels of the action are not coded); these criteria refer to “*slight*” appearances and would be coded as intensity level 1. When the action intensity exceeds the “slight” appearance, decide if it is “*marked or pronounced*” (level 2) or “*severe, extreme, or maximum*” (level 3).

***Symmetry*:** Every time AU10 or AU14 are coded, you will be prompted for an additional symmetry code:

- a: Asymmetrical/unilateral
- s: Symmetrical

Note: If any other AU’s are markedly asymmetrical, make a note of it in the comments area when coding. Otherwise, symmetry will be presumed.

**Other DFACS Codes**

| **Code** | **Key** | **Description** |
| --- | --- | --- |
| **Cannot code upper face** | **00** | Use this code if you cannot code the upper face. This may occur if the video goes out of focus, if the participant covers their upper face, if the participant goes off the frame of the camera, or if turns away such that you cannot get a clear view of their upper face. This may also be used if the participant has features (e.g., bangs, glasses) that obscure the upper face so much so that you cannot get a clear view. |
| **Cannot code lower face** | **99** | Use this code if you cannot code the lower face. This may occur if the video goes out of focus, if the participant covers their lower face, if the participant goes off the frame of the camera, or if turns away such that you cannot get a clear view of their lower face. This may also be used if the participant has features (e.g., facial hair) that obscure the lower face so much so that you cannot get a clear view. |
| **Crying/tears** | **c** | Code when tears are clearly visible (either welling up in eyes or rolling down face). If you aren’t sure, don’t code it. |
| **Vocalization/speech** | **v** | Code when the subject is making a vocalization (e.g., talking; mouthing the words to a song should also be coded here). Use this code if the person is talking and names an emotion (e.g., “I’m so scared!”). |
| **Emotion-related sound** | **q** | Code only when subject is making sounds clearly related to an emotion (e.g., laughing, wailing, scoffing, awwww, screaming, etc.). |
| **Other mouth/facial movement** | **w** | Code when you see any movement of the mouth or face that was made but not associated with one of the specific AU’s designated in Pass 1 or Pass 2.  If something is identified as clearly a non-emotion facial movement (i.e., swallow, sneeze, cough, yawn), even if it includes identifiable AU’s, just code those movements as “other” and don’t code the AU’s. Example: If a bilateral 14 is determined to be a swallow, just code as “other.”  Note: Do not code blinks unless they are part of a tic, then code as tic (see below). |
| **Face/head/neck touching** | **e** | Subject touches any part of face/head/neck. |
| **Look away/ obscures vision** | **r** | Subject uses hand or object to block their view of the stimulus; subject distinctly averts gaze from the stimulus (e.g., looks at floor or looks away, clearly cannot see stimulus). Only code if it seems clear the stimulus is no longer in gaze path (i.e., if gaze only seems slight/possibly averted but screen likely in line of vision, don’t code). |
| **Tic** | **t** | If someone has an identifiable tic, code all movement associated with tic each time it happens *(and don’t code any other AU’s associated with the tic).* |
| **Marker** | **m** | Markers are used to denote the start and end of each segment to be coded. |

**DFACS Coding Process Guidelines**

You will view each video segment three times:

**FIRST PASS: Lower Face AUs**

- Watch the video at either ***1/5 or 1/2 speed*, audio muted*.**
- Look for any activity in the lower face.
- Whenever activity is observed, activate the relevant lower face AU(s); deactivate when AU ends.
- All AU’s require a modifier code indicating intensity (1 to 3 scale); some also will require a symmetry modifier.
- Enter comments as needed.

**SECOND PASS: Upper Face AUs**

- Watch the video at ***1/5 or 1/2 speed*, audio muted*.**
- Look for any activity in the upper face.
- Whenever activity is observed, activate the relevant upper face AU(s); deactivate when AU ends.
- All AU’s require a modifier code indicating intensity (1 to 3 scale).
- Enter comments as needed.
- If you notice any issues with your lower face coding as you do this viewing, you can correct it.

**THIRD PASS: All Other Codes**

- Watch the video at either ***1/2 or*** ***full speed, audio on*.**
- Add any “other” codes as needed (e.g., non-emotion facial movements, vocalization, etc).
- Enter comments as needed.
- Use this pass to review that no scoreable upper or lower face actions have been missed, and to check for accuracy in your completed coding.
- If you notice any issues with your previous coding as you do this viewing, you can correct it.

*Speed up to the discretion of the coder based on what helps them optimally capture all movements for that participant.

**General Coding Guidelines**

- ***Code to the Letter of the Manual***. That is, only code an AU if it meets the criteria defined in the FACS manual. If it doesn’t conform to the criteria (or you aren’t sure if you are seeing an AU), then don’t code it. For anything you code, you need to be able to justify your coding decision (i.e., be able to state why it met the criteria for a given FACS code). Reminder: look for appearance changes that are unique when deciding to code an AU that shares features with another present AU.
- ***When in Doubt, Leave it Out.*** If you are uncertain if something occurred, don’t code it. This rule applies when (a) determining whether an AU acted or not, (b) determining if an AU incremented with other AUs that acted, (c) determining if an AU incremented or decremented in intensity. Thus, only the most obvious aspects of the activity are scored. Bottom line: if something is so unclear that you aren’t sure it is happening, DO NOT CODE IT.
- ***Take Notes.*** Some codes (e.g., non-emotion codes) will automatically prompt you to enter notes, though most AU’s will not. Please take as many notes as possible, indicating why you selected the code you did. This is particularly important in cases where you were torn between two options (e.g., AU9 vs AU10, or an AU vs. a non-emotion code). Specify what made you make your decisions, noting as many specific details as possible. *Mandatory notes:* always make a comment when you observe a notable asymmetry in an AU.
- ***Keep it Moving.*** Pause as needed to enter codes and pinpoint onsets/offsets, but try to keep the coding moving along. If you aren’t sure about how to code something, you are allowed to review segments of the clip a maximum of three times, then make your decision and move on.
- ***Use Your Tools***. When coding, you should ALWAYS have 1) the DFACS manual, 2) the FACS manual, and 3) a hand mirror. If you are having trouble deciding a code, remember to:
  - Use your own face -- model the AUs you are choosing between to see which looks more like the behavior you are coding
  - Try viewing the stimulus backwards and forwards in both slow motion and real time to pinpoint muscle movements (as well as onset and offset)
  - Use the manuals. Check the FACS manual for the AU movement and the intensity requirements to make sure your decision is justified. Consult the subtle difference tables and sample images in the FACS manual as needed to help with additional clarification.
- ***Get the Neutral Image Right***. Good coding requires a good neutral image. Take the time required to determine a neutral image you feel confident about; this will be done by pairs of coders who have to agree on the neutral. Once that neutral image is established, all coders MUST use it for that subject. Never create your own and code based on a different neutral than was used by other coders. If you disagree strongly with the established neutral, work with the other coder to select a new image. Then all coding must be done with the new image as the neutral baseline. Reminder: coders must study the neutral image before beginning coding.

# Observer Set Up

**Definitions**

Observer XT: The program which records observations; also allows for coding/analysis of collected data.

Project: A project is the folder system created by Observer to sync all coding, physio and video files. You will access project files in Observer for coding videos.

Observation: The observation is a container of data and video/audio files, not a file itself.

Event Log: The file containing data scored manually or imported into the Observer XT program.

PIDN: All participants have a ID number; this is the unique identifier for the participant at the MAC.

Mutually exclusive variable: This is the variable type in which only one variable may be selected at a time. We are not using these variables in DFACS.

Start-stop variable (state events): This variable type is for all AU codes and non-emotion codes. They can co-occur, and each need to be started and stopped independently.

Modifiers: This is the additional information need when input a variable. For example, this is the intensity level for AU codes (1, 2, or 3).


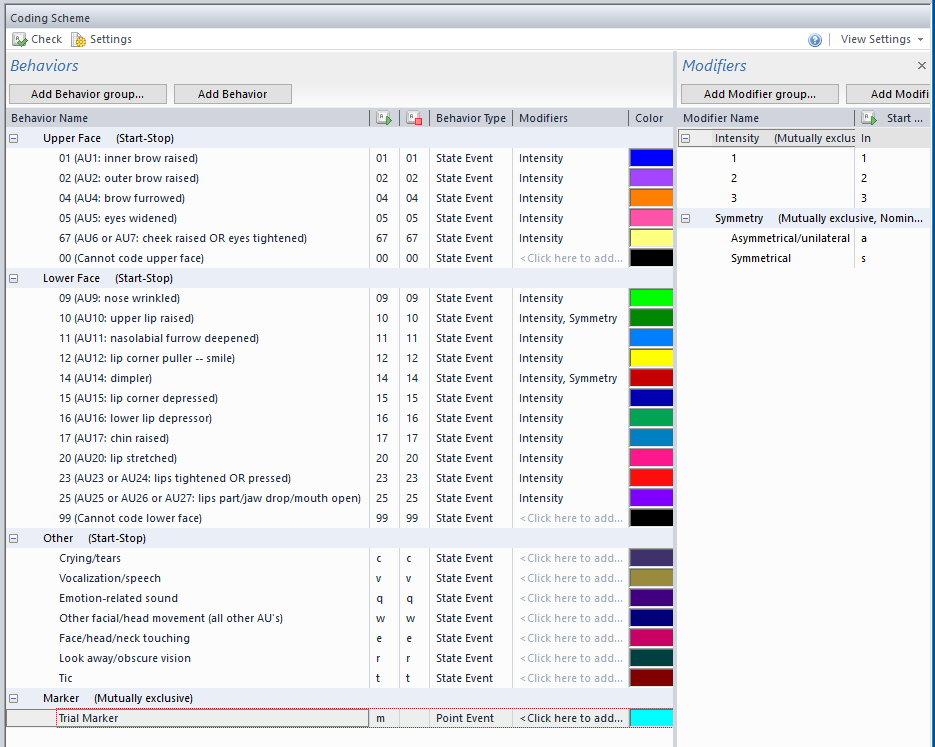
**Observer interface:**

Note: the codes are listed according to four types.

- **Upper face:** 5 upper face AU codes, as well as one option if the upper face cannot be coded.
- **Lower face:** 11 upper lower AU codes, as well as one option if the lower face cannot be coded.
- **Other:** All other codes aside from the designated AU’s. This includes other AU’s, non-emotion movements (e.g., swallowing, coughing), as well as behaviors such as vocalizations, looking away, or face touching.
- **Trial Markers:** Point event markers are used to indicate the start and stop of each trial to code.

# DFACS Reliability Notes – General Guidelines

--Coders are research assistants in the REH lab; all coders are FACS trained and certified prior to starting this project.

--Coders are blind to study hypotheses or diagnostic condition of any of the subjects they are coding.

--Coders use the Noldus Observer software program to do the coding. The coding system was designed so that coders hit a key (s) when the target movements begins, and entered the keys again when the target movements stops (or changes intensity). Any combination of the codes can be present at a given moment of time; all codes for all target movements are start and stopped independently.

--Coders practiced on videos from an unrelated project until they were comfortable with the coding system and adequate reliability was achieved.

-- Coders are assigned videos each week and code on their own. Coders meet as a team once per week to review codes and go over any inter-coder disagreements.

--Following the guidance of Cohn, Amabadar, and Ekman (2007), “We define reliability as agreement between two or more observers about the occurrence, intensity, and timing of action units.” As they further note, “Agreement is a more stringent measure [than summary counts] in that coders must not only be consistent about the number of times an action unit occurs but must also agree on when each one occurred.” These authors recommend quantifying agreement between coders using coefficient kappa.

--Accordingly, we calculate inter-observer agreement with coefficient kappa, which is the proportion of agreement above what would be expected to occur by chance (Cohen, 1960; Fleiss, 1981). Coefficients of 0.60 to about 0.75 indicate good, or adequate reliability; coefficients of 0.75 or higher indicate excellent reliability (e.g., Fleiss, 1981).

--Kappa coefficients can be calculated in two ways:

1. *Second-by-second coder agreement*. We exported coder data on a second-by-second basis. If an AU was activated during a given second of time, the data shows a 1. If it was not activated, the data shows a 0. Data from the two coders for all upper face and lower face AUs over all seconds of coded data were entered into a confusion matrix to generate a kappa coefficient indicating agreement about the number of times an action unit occurred as well as when it occurred. This approach is useful when specifically focusing on the level of agreement between coders on the presence and timing of FACS-defined action units.
2. *
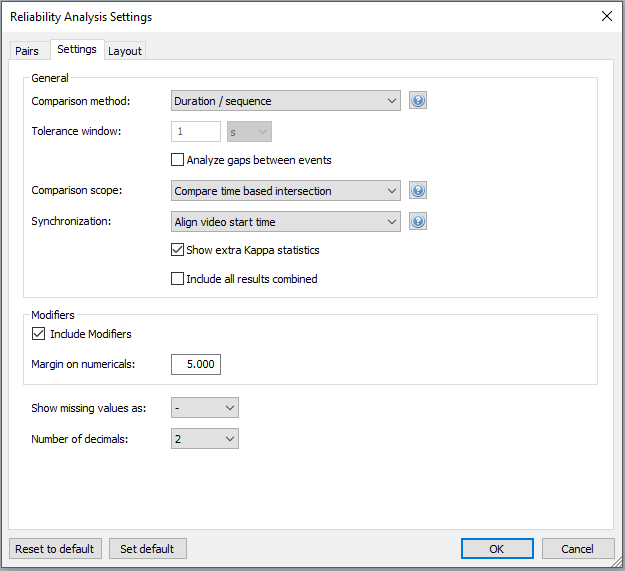
Using the Noldus system.* For a more comprehensive estimate of reliability that factors in level of agreement on intensity levels, we used the reliability analysis function within the Observer system. The following settings should be selected within the Observer system when running the reliability analysis. It directs the calculation to compare state events (i.e., the moments when an emotion was coded) as a function of the duration of overlap and non-overlap. The reliability analysis generates a confusion matrix showing the degree of agreement between two coders for all codes within the system (factoring in agreement on modifiers), with the time frame at the hundredth of a second.

# Source Material for DFACS

**PRIMARY SOURCE:** All criteria for coding upper and lower face action units come directly from the FACS manual.

Ekman, P., Friesen, W.V., & Hager, J.C. (2002). *Facial Action Coding System: The Manual on CD ROM*. Salt Lake City, UT: Network Information Research Corporation.

**Additional Source Materials:**

Barrett, L. F., Adolphs, R., Marsella, S., Martinez, A. M., & Pollak, S. D. (2019). Emotional expressions reconsidered: Challenges to inferring emotion from human facial movements. *Psychological science in the public interest*, *20*(1), 1-68.

Campos, B., Shiota, M.N., Keltner, D., Gonzaga, G.C., & Goetz, J.L. (2013). What is shared, what is different? Core relational themes and expressive displays of eight positive emotions, Cognition and Emotion, 27(1), 37-52.

Cohn, J. F., Ambadar, Z., & Ekman, P. (2007). Observer-based measurement of facial expression with the Facial Action Coding System. *The handbook of emotion elicitation and assessment*, 203-221.

Cordaro, D. T., Sun, R., Keltner, D., Kamble, S., Huddar, N., & McNeil, G. (2017). Universals and cultural variations in 22 emotional expressions across five cultures.

Du, S., Tao, Y., & Martinez, A. M. (2014). Compound facial expressions of emotion. *Proceedings of the National Academy of Sciences*, *111*(15), E1454-E1462.

Ekman, P., Friesen, W.V., & Hager, J.C. (2002). *Facial Action Coding System: Investigator’s Guide*. Salt Lake City, UT: Network Information Research Corporation.

Ekman, P., Irwin, W., & Rosenberg, E. (1994). *EMFACS: CODERS INSTRUCTIONS (EMFACS-8)*. Unpublished manuscript.

Ekman, P., Matsumoto, D., Friesen, W.V. (1997). Facial expression in affective disordrers. In P. Ekman & E.K. Rosenberg (Eds) What the face reveals. New York: Oxford University Press.

Gottman, J., Levenson, R., & Woodin, E. (2001). Facial expressions during marital conflict. *Journal of Family Communication*, *1*(1), 37-57.

Keltner, D., & Cordaro, D. T. (2017). Understanding multimodal emotional expressions. *The science of facial expression*, 1798.

Keltner, D., Sauter, D., Tracy, J., & Cowen, A. (2019). Emotional expression: Advances in basic emotion theory. *Journal of nonverbal behavior*, *43*(2), 133-160.

Kring, A.M. & Sloan, D. The Facial Expression Coding System (FACES): A Users Guide. Unpublished manuscript.

Lucey, P., Cohn, J. F., Kanade, T., Saragih, J., Ambadar, Z., & Matthews, I. (2010, June). The extended cohn-kanade dataset (ck+): A complete dataset for action unit and emotion-specified expression. In *Computer Vision and Pattern Recognition Workshops (CVPRW), 2010 IEEE Computer Society Conference on* (pp. 94-101). IEEE.

Matsumoto, D., Ekman, P., & Fridlund, A. (1991). Analyzing nonverbal behavior. In Dorwick, P.W. (Ed.) Practical guide to using video in the behavioral sciences (p. 153-165). New York: Wiley & Sons.

Matsumoto, D. & Ekman, P. (2008). Facial expression analysis, *Scholarpedia, 3*(5), 4237.

Matsumoto, D., Keltner, D., Shiota, M. N., O’Sullivan, M., & Frank, M. (2008). Facial expressions of emotion. *Handbook of emotions*, *3^rd^ Edition*, 211-234.

Rosenberg, E. L., Zanesco, A. P., King, B. G., Aichele, S. R., Jacobs, T. L., Bridwell, D. A., MacLean, K. A., Shaver, P. R., Ferrer, E., Sahdra, B. K., Lavy, S., Wallace, B. A., & Saron, C. D. (2015). Intensive meditation training influences emotional responses to suffering, *Emotion*, *15*(6), 775-790.

Sayette, M. A., Cohn, J. F., Wertz, J. M., Perrott, M. A., & Parrott, D. J. (2001). A psychometric evaluation of the facial action coding system for assessing spontaneous expression. *Journal of Nonverbal Behavior*, *25*(3), 167-185.

Tian, Y. I., Kanade, T., & Cohn, J. F. (2001). Recognizing action units for facial expression analysis. *IEEE Transactions on pattern analysis and machine intelligence*, *23*(2), 97-115.
