# Supplementary material for: Structural neuroanatomy of human facial behaviors
Source: Soc Cogn Affect Neurosci. 2024 Sep 23;19(1):nsae064. doi: 10.1093/scan/nsae064 (PMC11492553; doi:10.1093/scan/nsae064)
Supplement: nsae064_Supp [file nsae064_supp.zip › scan-23-296-File011.docx]

**Table S1. Functional Anatomy of the Coded Facial Action Units.** We coded 16 action units (AUs) in the video recordings of the participants using the Dynamic Facial Action Coding System.

| AU | Function | Muscle(s) |
| --- | --- | --- |
| AU 1 | Inner Brow Raiser | *Frontalis, pars medialis* |
| AU 2 | Outer Brow Raiser | *Frontalis, pars lateralis* |
| AU 4 | Brow Lowerer | *Corrugator supercilii* |
| AU 5 | Upper Lid Raiser | *Levator palpebrae superioris* |
| AU 6/7 | Cheek Raiser/Lid Tightener | *Orbicularis oculi, pars orbitalis/ pars palpebralis* |
| AU 9 | Nose Wrinkler | *Levator labii superioris alaquae nasi* |
| AU 10 | Upper Lip Raiser | *Levator labii superioris* |
| AU 11 | Nasolabial Deepener | *Zygomaticus minor* |
| AU 12 | Lip Corner Puller | *Zygomaticus major* |
| AU 14 | Dimpler | *Buccinator* |
| AU 15 | Lip Corner Depressor | *Depressor anguli oris* |
| AU 16 | Lower Lip Depressor | *Depressor labii inferioris* |
| AU 17 | Chin Raiser | *Mentalis* |
| AU 20 | Lip stretcher | *Risorius* |
| AU 23/24 | Lip Tightener/Lip Presser | *Orbicularis oris* |
| AU 25 | Lips part | *Depressor labii inferioris* |
