# Supplementary material for: Structural neuroanatomy of human facial behaviors
Source: Soc Cogn Affect Neurosci. 2024 Sep 23;19(1):nsae064. doi: 10.1093/scan/nsae064 (PMC11492553; doi:10.1093/scan/nsae064)
Supplement: nsae064_Supp [file nsae064_supp.zip › scan-23-296-File012.docx]

**Table S2. Definitions and Computations of Facial Behavior Measures.**

|  | **Variable Definition** | **Description** |
| --- | --- | --- |
|  |  |  |
| **Within-Trial Measures** |  |  |
| AU Combination Activity | ${AU}_{i,j (trial)}= \sum_{t=1}^{T} \frac{{AU}_{i}\left( t \right)}{T} + \sum_{t=1}^{T} \frac{{AU}_{j}\left( t \right)}{T} , T=30$ | The second-by-second intensity scores for AUs 12 & 6/7 in the amusement trial and AUs 4 & 6/7 and AUs 9 & 10 in the disgust trial, averaged over 30 seconds in each trial, were summed to obtain context-specific measures of AU combination total activity scores within a trial. |
|  |  |  |
|  |  |  |
| **Across-Trials Measures** |  |  |
|  |  |  |
| AU Combination Activity | ${AU}_{i,j (total)}= \sum_{t=1}^{T} \frac{{AU}_{i}\left( t \right)}{T} + \sum_{t=1}^{T} \frac{{AU}_{j}\left( t \right)}{T} , T=150$ | The second-by-second intensity scores for AUs 12 & 6/7 and AUs 4 & 6/7, averaged over 150 seconds, were summed to obtain measures of AU combination total activity scores across the five trials. |
| Single AU Activity | ${AU}_{i (total)}=\sum_{t=1}^{T} \frac{{AU}_{i}\left( t \right)}{T} , T=150$ | The second-by-second intensity scores for each AU were averaged across the five trials, which provided a single AU activity score for each AU over 150 seconds. |
|  |  |  |
| Total Facial Behavior | ${AU}_{(total)}= \sum_{i=1}^{N} \sum_{t=1}^{T} \frac{{AU}_{i}\left( t \right)}{T} , T=150 \& N=16$ | The total activity scores of the 16 AUs were summed to obtain a measure of total facial behavior across the five trials. |

| *AU* = action unit | *AU_i (total)_* = Average intensity for the i^th^ AU over the duration of all trials |
| --- | --- |
| *T* = Total time in seconds | *AU _(total)_* = Sum of average intensities for all AUs over the duration of all trials |
| *N* = Total number of coded AUs | *AU_i,j (total)_* = Sum of average intensities for the i^th^ and ^jth^ AUs over the duration of all trials |
| *AU_i_ (t)* = Intensity of the i^th^ AU at time t (second) | *AU_i,j (trial)_* = Sum of average intensities for the i^th^ and ^jth^ AUs over the duration of a given trial |
|  |  |
