# Supplementary material for: Structural neuroanatomy of human facial behaviors
Source: Soc Cogn Affect Neurosci. 2024 Sep 23;19(1):nsae064. doi: 10.1093/scan/nsae064 (PMC11492553; doi:10.1093/scan/nsae064)
Supplement: nsae064_Supp [file nsae064_supp.zip › scan-23-296-File013.docx]

**Table S3. Total Activity in Action Units Within and Across Trials.** We calculated a total activity score for each AU by averaging the intensity scores within each trial (30 seconds) and across trials (150 seconds). Here, “All AUs” refers to the sum of all AU activity in each trial, and “All Trials” refers to the total activity in each AU across trials. The most intense facial behavior occurred during the amusement and disgust trials, emotions that are easier to elicit in the laboratory and more difficult to suppress (Gross & Levenson, 1993; Giuliani et al. 2008).

|  | Awe | Sadness | Amusement | Disgust | Nurturant Love | All Trials |
| --- | --- | --- | --- | --- | --- | --- |
| AU 1 | 0.16 | 0.59 | 0.10 | 0.27 | 0.14 | 0.25 |
| AU 2 | 0.16 | 0.18 | 0.11 | 0.10 | 0.08 | 0.12 |
| AU 4 | 0.17 | 0.62 | 0.05 | 1.38 | 0.23 | 0.49 |
| AU 5 | 0.04 | 0.15 | 0.04 | 0.05 | 0.05 | 0.06 |
| AU 6/7 | 0.47 | 0.50 | 2.23 | 1.18 | 1.51 | 1.17 |
| AU 9 | 0 | 0 | 0 | 0.32 | 0 | 0.06 |
| AU 10 | 0 | 0 | 0.04 | 0.24 | 0.03 | 0.06 |
| AU 11 | 0 | 0 | 0 | 0 | 0 | 0 |
| AU 12 | 0.34 | 0.17 | 2.08 | 0.19 | 1.33 | 0.82 |
| AU 14 | 0 | 0.01 | 0 | 0.04 | 0.01 | 0.01 |
| AU 15 | 0 | 0.01 | 0 | 0.04 | 0 | 0.01 |
| AU 16 | 0 | 0 | 0 | 0 | 0 | 0 |
| AU 17 | 0.11 | 0.16 | 0.07 | 0.09 | 0.13 | 0.11 |
| AU 20 | 0 | 0 | 0.03 | 0.02 | 0 | 0.01 |
| AU 23/24 | 0.06 | 0.03 | 0.07 | 0.12 | 0.05 | 0.06 |
| AU 25 | 0.01 | 0.02 | 0.33 | 0.11 | 0.16 | 0.12 |
| All AUs | 0.1 | 0.15 | 0.32 | 0.26 | 0.23 | 0.2 |
