# Supplementary figures and images for: Structural neuroanatomy of human facial behaviors
Source: Soc Cogn Affect Neurosci. 2024 Sep 23;19(1):nsae064. doi: 10.1093/scan/nsae064 (PMC11492553; doi:10.1093/scan/nsae064)

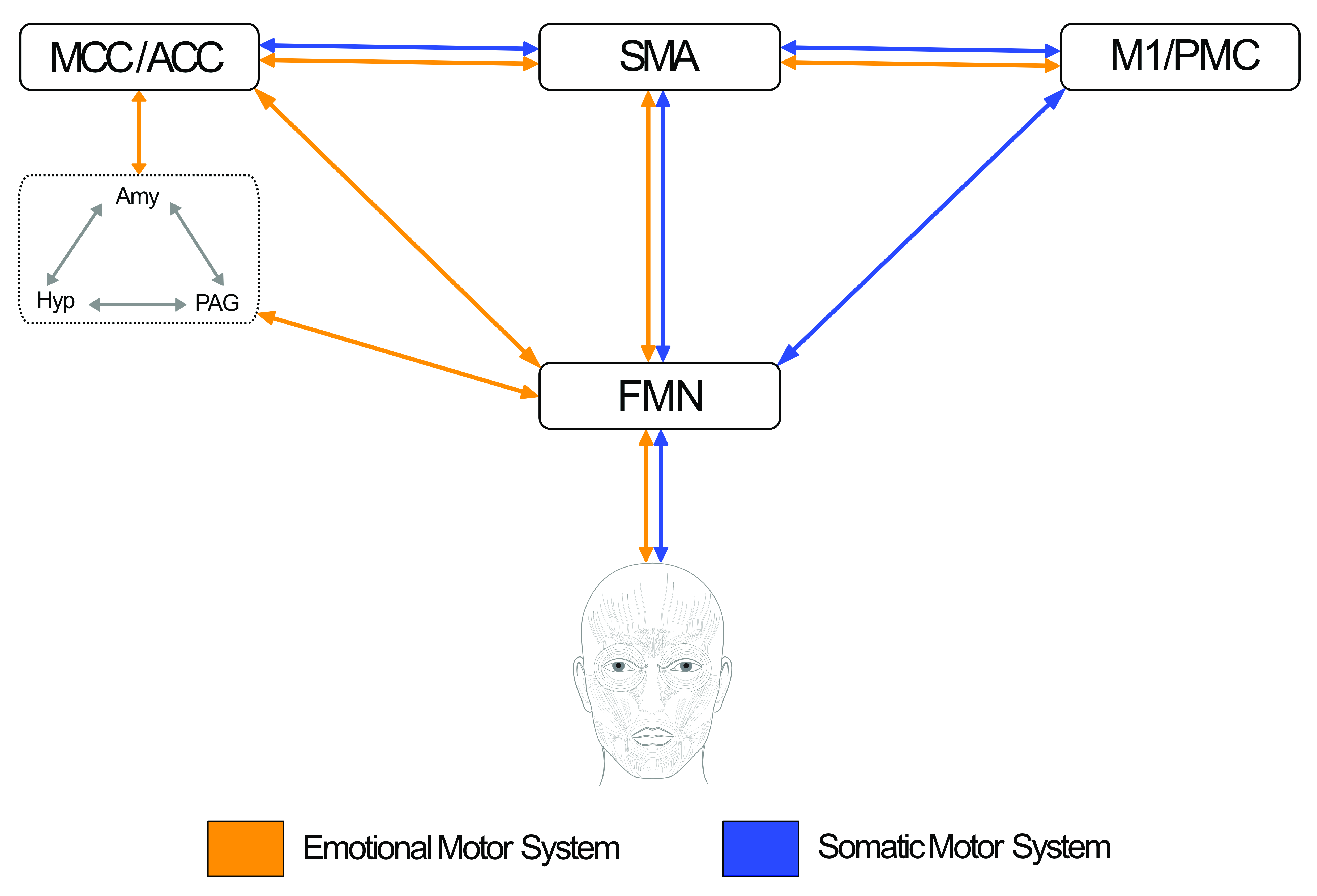

Supplement: nsae064_Supp [file nsae064_supp.zip › scan-23-296-File014.tiff]

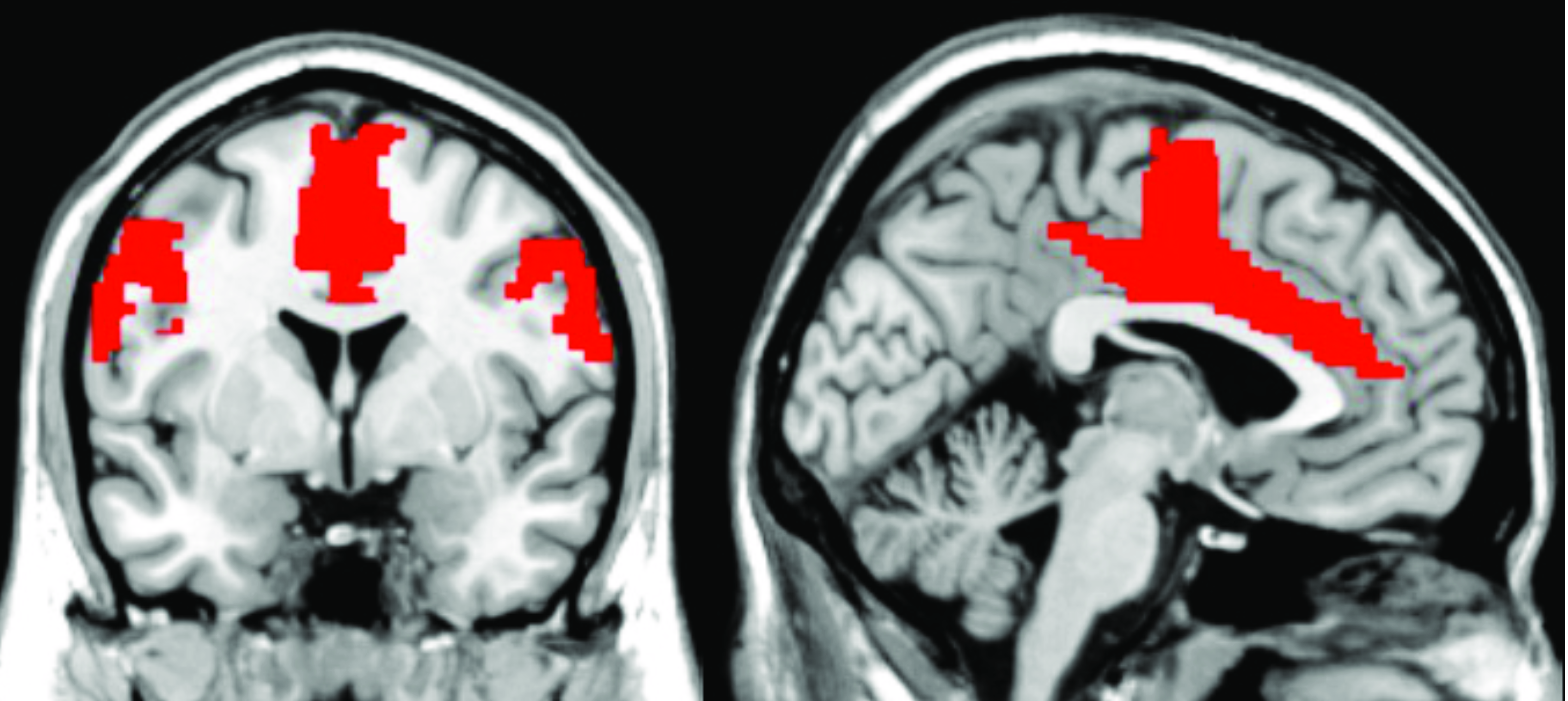

Supplement: nsae064_Supp [file nsae064_supp.zip › scan-23-296-File015.tiff]

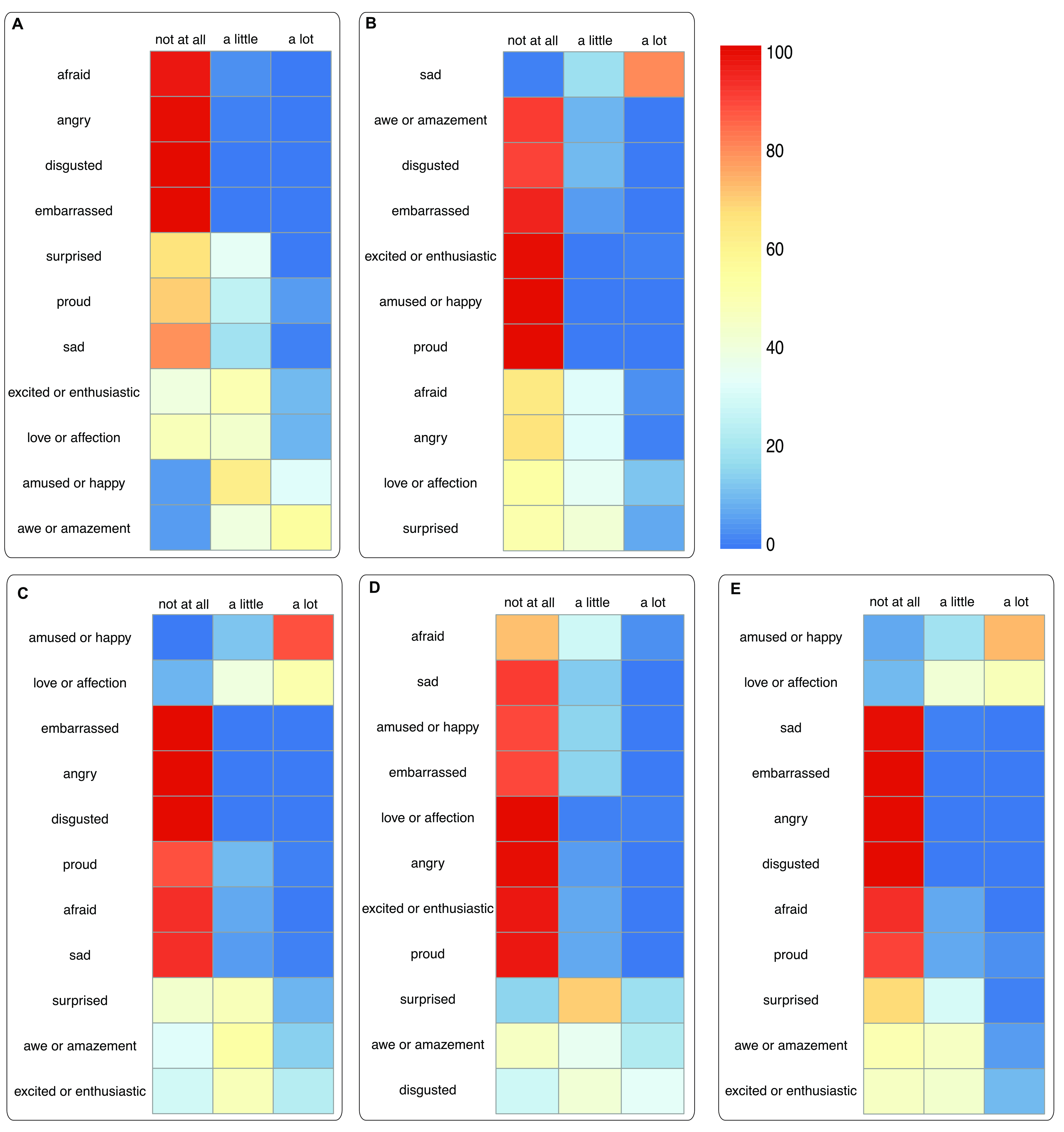

Supplement: nsae064_Supp [file nsae064_supp.zip › scan-23-296-File016.tiff]
